# Meeting Report from the Genomic Standards Consortium (GSC) Workshop 10

**DOI:** 10.4056/sigs.1423520

**Published:** 2010-12-25

**Authors:** Elizabeth Glass, Folker Meyer, Jack A Gilbert, Dawn Field, Sarah Hunter, Renzo Kottmann, Nikos Kyrpides, Susanna Sansone, Lynn Schriml, Peter Sterk, Owen White, John Wooley

**Affiliations:** 1 Argonne National Laboratory, Argonne, IL USA; 2 Computation Institute, University of Chicago, Chicago, IL USA; 3 Department of Ecology and Evolution, University of Chicago, Chicago, IL USA,; 4 Centre for Ecology & Hydrology, Oxfordshire, UK; 5 European Molecular Biology Laboratory (EMBL) Outstation, European Bioinformatics Institute (EBI), Hinxton, Cambridge UK; 6 Microbial Genomics Group, Max Planck Institute for Marine Microbiology and Jacobs University Bremen, Bremen, Germany; 7 DOE Joint Genome Institute, Walnut Creek, CA, USA; 8 University of Oxford, Oxford e-Research Centre, Oxford, UK; 9 Institute for Genome Sciences, University of Maryland School of Medicine, Baltimore, MD USA; 10 Wellcome Trust Sanger Institute, Wellcome Trust Genome Campus, Hinxton, Cambridge UK; 11 University of California San Diego, La Jolla, CA USA

## Abstract

This report summarizes the proceedings of the 10th workshop of the Genomic Standards Consortium (GSC), held at Argonne National Laboratory, IL, USA. It was the second GSC workshop to have open registration and attracted over 60 participants who worked together to progress the full range of projects ongoing within the GSC. Overall, the primary focus of the workshop was on advancing the M5 platform for next-generation collaborative computational infrastructures. Other key outcomes included the formation of a GSC working group focused on MIGS/MIMS/MIENS compliance using the ISA software suite and the formal launch of the GSC Developer Working Group. Further information about the GSC and its range of activities can be found at http://gensc.org/.

## Introduction

The Genomic Standards Consortium (GSC) formed in 2005 to tackle the challenge of working as a community towards improving the quantity and quality of contextual data made accessible for genomes and metagenomes [1]. The GSC works towards its goal through the creation, maintenance and adoption of a range of standards and collaborative projects. In 2008, the GSC also expanded its scope to include the description of marker gene sequences (i.e. ribosomal genes) [2]. Now, the GSC has created three **M**inimum **I**nformation checklists to cover contextual data for genomes, metagenomes and marker genes: Minimum Information about a Genome Sequence (MIGS), a Metagenome Sequence (MIMS) and an ENvironmental Sequence (MIENS) [3,4].

Currently, the GSC exists as an open-member international community consisting of 100+ biologists, bioinformaticians and computer scientists that includes representatives from EMBL, EMBL-EBI, DDBJ, NCBI and major sequencing centers including BGI, JCVI, JGI, and the Wellcome Trust Sanger Institute (WTSI). In addition to the core work of the GSC on the development of minimal information checklists [3,4] the GSC is also working towards adoption of these standards, including the launch of this journal, *Standards in Genomic Sciences* (SIGS) [5]. Implementation and adoption projects include the Genomic Contextual Data Markup Language (an XML data format to support GSC minimal standards) [6], the Genomic Rosetta Stone (a resolving service for top-level genome and metagenome project information from different resources) [7] and Habitat-Lite (a lightweight ecological ontology) [8]. The GSC is now working on how to cope with the large quantities of metagenomic data in the form of the M5 project [9] (http://gensc.org/gc_wiki/index.php/M5), the focus of this workshop.

The GSC builds consensus through the hosting meetings and working groups. The GSC 10 workshop was held on the campus of Argonne National Laboratory (ANL), which occupies 1,500 beautiful, wooded acres, about 25 miles southwest of Chicago and surrounded by the Waterfall Glen Forest Preserve. ANL is one of the  leading federally funded research and development centers in the US, and maintains a core metagenomics research group and sequencing facility. The workshop consisted of three focused working group meetings and two days of open sessions with presentations, discussions and break out groups.

## The ISA software suite workshop

The GSC meeting started on Oct 4th with a workshop dedicated to the use of the ISA software suite. While several key groups already interested in working with these tools formed the core of the meeting, the event was open to all and attendance was very high. The scope of this workshop was to provide an overview of the ISA Infrastructure tool kit and discuss how it can be used within the GSC to promote compliance with MIGS/MIMS/MIENS. This meeting was organized by Susanna-Assunta Sansone and Dawn Field. To start, **Susanna Sansone (University of Oxford)** gave an overview of the software suite and its role in data sharing. The Investigation/Study/Assay (ISA) tools ( [10]; ) offer a way to capture MIGS/MIMS/MIENS compliant metadata as well as metadata describing a range of other types of investigations. The ISA tools are freely available as a desktop software suite, targeted to curators and experimentalists, and empowers users by enabling better access to minimum information checklists and ontologies. This can be used to describe studies employing one or a combination of technologies and to submit that metadata to suitable public repositories, including the European Nucleotide Archive (ENA; genomics), PRIDE (proteomics) and ArrayExpress (transcriptomics).

Following this overview, **Phillipe Rocca-Serra** and **Eamonn Maguire (University of Oxford)** gave more detailed presentations on the actual use of the tools. Key outcomes of the discussions that followed included agreement to formalize an ISA working group within the GSC as part of its compliance activities.

## GSC Board meeting

The GSC established a board in April 2009 whose members have been selected from representatives within the wider community most active in driving GSC activities. The Board currently has 21 members and a set of standing committees. Membership in GSC standing committees is currently defined by participation and the Board welcomes anyone from the wider community that would like to join a standing committee. This Board meeting covered a range of topics, but focused primarily on how to continue the formalization of the GSC. Key outcomes include a decision to aim for two meetings a year, one of which is to be an open registration meeting designed to engage with the wider community (April time) and one smaller meeting focused specifically on progressing GSC projects.

## GSC Developer Meeting

In the afternoon the first face-face meeting of the GSC’s Developer working group took place. **Renzo Kottmann, (Max Planck Institute for Marine Microbiology, Bremen)** chair of this session and the Developer group, first described the goals of the group and progress to date and then opened the floor for discussion. The discussion allowed everyone to introduce himself or herself in person, give updates on their particular activities and therefore set the stage for the rest of GSC 10. The meeting was very well attended and the introductions attested to the wide range of adoption activities ongoing in the community. The role of this group is to push forward GSC projects on a technical level, and GSC contributions to other standardization projects, through technical discussions of the best solutions for implementation and work with adopters towards implementation of GSC standards. The GSC developer group was defined as an intersection between all the other GSC working groups. The main goal was achieved: the members got to meet each other and talk at length, and several new members joined. The group will communicate mainly *via* the newly established mailing list (see http://lists.gensc.org/mailman/listinfo/gensc-developers for details on subscription). Additionally, the RCN4GSC grant finances monthly teleconference calls. The membership is defined by participation so new members are encouraged to consider joining the web page of the GSC Developer group is in the GSC wiki at http://gensc.org/gc_wiki/index.php/Developer_Group.

## Formal Opening of GSC 10 and Keynote talk on the Microbial Earth project

The formal start of GSC 10 came with the evening mixer followed by a Keynote talk on Microbial Earth by **Nikos Kyrpides (DOE Joint Genome Institute).** The Microbial Earth project aims to generate a comprehensive genome catalog of all the bacterial and archaeal type strains, currently estimated to be nearly 9,000 strains. It builds on the hundreds of stains currently being completed within the *Genomic Encylopedia of Bacteria and Archaea* project (GEBA) [11]. As the GEBA project demonstrates, we have only begun to scratch the diversity of microbial life and genome sequences are the ideal foundation for a wide range of downstream studies, including metagenomic studies of the environment.  Kyrpides emphasized that cost of DNA sequencing is no longer the bottleneck in realizing this dream for microbiology, and that there is enough capacity to complete the project within the next 2-3 years. The hope, however, is that this project would be pursued as an international effort, under the auspices of the GSC, thus developing and implementing GSC-driven genome standards on all aspects of the project.

## Day 2 - Session I: GSC Updates

Day 2 opened with a traditional session containing updates on all GSC project. **Rick Steven (Argonne National Laboratory)** gave a short welcome presentation, confirming the commitment of ANL to supporting the work of the GSC. This was followed by a short introduction to the GSC by current President, **Dawn Field (NERC Centre for Ecology and Hydrology). Folker Meyer (ANL)** covered the M5 project, **Peter Sterk (GSC)** covered the MIGS/MIMS/MIENS family of standards [3,4], **Renzo Kottmann **covered GCDML [6], Wim deSmet covered the Genomic Rosetta Stone [7] and **Norman Morrison (University of Manchester)** discussed efforts to use ontologies within the GSC. These core project updates were followed by three talks on building community links within the GSC. **Brian Bramlett (Lux Bio)** described plans to create a partnership between the GSC and the future Digital Biology Foundation, **Susanna Sansone **described the role of the GSC as a founding community in the BioSharing forum, and **Norman Morrison **described progress towards the formation of the Biodiversity working group within the GSC.

### Session II: Adoption of GSC recommended standards

The second morning session gave the floor to speakers from a variety of adoption activities, and was chaired by **Nikos Kyrpides, Renzo Kottmann, **and **Dawn Field ** formally introduced the work of the developers group. **David Aanensen (Imperial College, London)** talked about compliance with GSC standards through SMART phones. Specifically, David gave an overview of his Epicollect system [12]. **James Cole (Michigan State University)** talked about efforts by the RDP project to support submission of MIENS compliant data and **Philippe Rocca-Serra ** talked about configurations to the ISA software tools to suport MIGS/MIMS/MIENS compliance and the use of ontologies.

**Lynn Schriml (University of Maryland)** then gave an overview of the GSC Research Coordination Network. Funding for this RCN4GSC project contributes to some costs of the GSC workshops and in particular supports exchange visits between laboratories. To date both Renzo Kottmann and Eamonn Maguire have completed such exchange visits and the GSC is now looking for further nominees. **Owen White (University of Maryland)** gave an update on the CAFAE proposal, an effort in which GSC would be closely involved. On behalf of **George Garrity (Michigan State University), Peter Sterk (GSC)** then gave an update on the eJournal of the GSC, *Standards in Genomic Sciences* (SIGS). Since its launch in July 2009 until September 2010, 97 papers had been published online. Of those the majority (81) were short genome reports. In addition, there were six Standard Operation Procedures (SOPs), five meeting reports, four editorials, three research articles, one white paper, one community dialog and two *errata*. SIGS ranked fourth place in the top ten of journals publishing genome reports based on data obtained from the GenomesOnline database on September 27, 2010 [13].

### Session II: The Vision of M5

The opening session was chaired by **Owen White **and contained a series of talks outlining the technologies that would likely play a key role in the realization of a future M5 collaborative platform. Owen White set the tone of the session by urging all presenters to develop a set of specific milestones for the initialization of M5. The milestones, if achieved over the next six months, would result in the initial infrastructure for sharing large data sets among genome centers, allow computational search results to be more reusable, and to promote cloud-based analyses on these data.

**Andreas Wilke (Argonne National Laboratory)** then described the current status of the first M5 pilot project, the creation of a file format for exchanging processed metagenomic data. This will help reduce recomputation and enable the composition of the best components from existing pipelines The Metagenomics Transfer Format (MTF) will consist of raw sequence data, as well as transformed sequences, feature coordinates (“gene finding”), similarities, metadata and workflow description (“provenance”). Preliminary work will be done in conjunction with Nikos Kyrpides’ team in order to define detailed implementation of the exchange format.

**Sarah Hunter (European Bioinformatics Institute)**, the co-chair of the M5 working group, gave some background on the purpose of the ELIXIR effort, which is to build a plan for a sustainable infrastructure for biological information in Europe. She then discussed the importance of M5 to users and the responsibilities that data and infrastructure providers have to these users. In brief, those responsibilities are: (1) to ensure that users do not need to invest in new computer hardware to run analyses; (2) to create curated workflows; (3) to have standardized interfaces and formats; (4) to store all necessary information so that  users may be able to interpret data correctly, independent of its source; (5) to avoid re-running analyses and (6) promote interoperability by adopting standards. She argued that these can only be achieved through collaboration and presented some ideas and points for further discussion.

The focus then shifted to talks on the promise of cloud computing and. **Sam Anguioli (University of Maryland)** spoke about the CLOud Virtual Resource (CLOVR) project, which aims to put genomic pipelines in the cloud by building a CLOVR virtual machine. **Jared Wilkening (Argonne National Laboratory)** discussed parallelizing CLOVR in clouds and clusters with AWE (‘Another Workflow Engine’). **Jeff Grethe** (**University of California San Diego)** then shifted the topic to workflows and described how workflows  are being used in the context of the CAMERA project [14]. **Ilkay Altintas (University of California San Diego)** followed up with a discussion the use of the Kepler workflow tool [15] in the CAMERA project and beyond. **Katy Wolstencroft (University of Manchester)** closed the session with a talk on Taverna, another workflow tool. [15] and the MyExperiment project [16] which aims to archive workflows for use by the wider community.

### Session IV: M5 and coordinated megasequencing projects

Following the technical presentations on technologies suitable for building the M5 platform, the focus switched to the wealth of data that will be generated in the near future. The session was chaired by **Hans-Peter Klenk (DSMZ)** and included descriptions of four additional megasequencing projects. An *ad hoc* definition of a megasequencing project is a project that generates more than 500 billion base pairs of sequencing or analyzes more than 100 samples. **Folker Meyer **gave a brief update on discussions with the GSC at the Sloan indoor metagenomics meeting held earlier in the year. This programme aims to understand the microbes of man-made spaces (i.e. within building). An action of this meeting was for participants to elaborate an ‘indoor spaces’ package within the MIENS list of environmental packages. **Jack Gilbert (Argonne National Laboratory)** gave an overview of the community proposal for an Earth Microbiome Project (EMP; www.earthmicrobiome.org), which aims to sequence more than 100,000 environmental samples from a range of environments across the globe. Pilot studies for this initiative are now ongoing, but the aim is to produce more than 5 petabase pairs of information by 2014. This project was an outcome of the Terabase Metagenomics (http://icis.anl.gov/programs/summer-1) meeting held earlier in the year. Jack Gilbert also filled in for the Beijing Institute for Genomics (BGI) representative, providing an overview their astounding sequencing capacity and their work towards the 1000 genomes project and beyond.

The formal session was closed the interested parties dispersed to attend break out groups where technical demonstrations of two relevant software platforms were demonstrated. **Narayan Desai (Argonne National Laboratory)** gave a live demo of the Magellan platform for bio-computing and Philippe Rocca-Serra and Eamonn Maguire gave a live demo of the ISA software suite and how it supports standards compliance.

### Dinner and Plenary talk by Oliver Ryder on Genome 10k Project

Following another group dinner, **Oliver Ryder (San Diego Zoo)** gave the second Keynote talk of the meeting. Again describing another ambitious megasequencing project, he gave a vision of what the future will look like after we have completed 10,000 Vertebrate Genomes. This may sound like a large number, but it is still only one species per genus of extant vertebrate organisms. The Genome 10k Project has published a call to action in the Journal of Heredity [17] and is actively looking for sponsors to help take this work forward. This consortium has assembled a collection of 16,203 representative vertebrate species spanning evolutionary diversity across living mammals, birds, nonavian reptiles, amphibians, and fishes (ca. 60,000 living species). Sequencing these genomes will give unparalleled insights into vertebrate genome evolution, the tree of life, and a myriad of other fundamental scientific questions as well as aiding in the conservation of many of these species. The plan to sequence the genomes of the first 101 genomes by The Genome 10K Community of Scientists and BGI of Shenzhen, China was announced in December 2010 (http://www.cbse.ucsc.edu/news-article?ID=1820).

## DAY 3 - Session V: M5 - Building the roadmap

**Rob Knight (University of Colorado)** started off the third day of the meeting with an excellent talk entitled *The promise of metadata for science*. He gave an overview of a wide range of studies of microbial communities, including the human microbiome, in which access to contextual data formed a core part of the analysis and interpretation of the data. Through this presentation, Rob helped to underscore the value of GSC efforts and establish a vision for what needs to be done over the coming decade. Following this ‘charge’ talk, the focus of the meeting shifted back to discussions of the M5 roadmap.

### Session VI: Discussion of M5 and break out groups

Discussions about the M5 roadmap led to the decision to form break out groups to discuss the three key pilot projects. Folker Meyer led a discussion of the future of the Metagenomics Transfer Format (MTF) project. **Sam Anguioli **led a discussion on the feasibility of building a GSC M5 virtual machine based on CLOVR and the NEBC Bio-Linux package repository [18] and containing key pieces of software like QIIME [19] and the MG-RAST client software. Owen began exploration of a search application programming interface (API) that would allow integration of results. Upon coming back into plenary session, the M5 roadmap was discussed and the following key steps agreed upon.

In building the M5 roadmap, five key steps were identified. The first was to identify further collaborators to bring the required skills and technologies to the table to build M5. The second was to visualize the landscape of technologies that would be used within M5 (workflows, virtual images, clouds and instances of each where collaboration had been agreed) to build a ‘bird’s eye view’ of the full structure of a future M5 platform. The third was to reach explicit agreements on how the collaborators would work together and how further pilot projects would be developed. The fourth was the definition of key pilot projects, which include 1) MTF, 2) a GSC virtual machine image and 3) an API to access to a generic data model that would support queries in an M5 context. The latter two were agreed upon at GSC 10. Another suggested pilot project was a GSC repository of workflows and is already in progress, primarily between the Kepler and Taverna teams. Finally, in order to clearly communicate the vision and potential of the M5 platform, the group agreed that it will be necessary to draft a joint document.

### Wrap up and Actions

Dawn Field led a final round up of actions before closing the meeting. She polled the meeting participants on what they thought the top outcomes of the meeting were.

**The top answers were**
1. Advance M5, based on the three formally defined pilot projects
2. Formally launch the Developer group (first face-to-face meeting, recruitment)
3. Continue holding annual meetings (GSC 11 and 12 planning underway)
4. Encourage implementation and adoption of the GSC family of MIGS/MIMS/MIENS checklists
5. Adopt the new logo package designed by Eamonn Maguire for the GSC

To formally close the workshop, Dawn thanked all the organizers, sponsors, speakers and attendees.
